# Does age really matter? Radiotherapy in elderly patients with glioblastoma, the Munich experience

**DOI:** 10.1186/s13014-017-0809-9

**Published:** 2017-04-28

**Authors:** Christoph Straube, Hagen Scherb, Jens Gempt, Stefanie Bette, Claus Zimmer, Friederike Schmidt-Graf, Jürgen Schlegel, Bernhard Meyer, Stephanie E. Combs

**Affiliations:** 1 Department of Radiation Oncology, Klinikum rechts der Isar, Technische Universität München (TUM), Munich, Germany; 2 Deutsches Konsortium für Translationale Krebsforschung (DKTK), Partner Site Munich, Munich, Germany; 3 Institute of Computational Biology, Helmholtz Zentrum München, Deutsches Forschungszentrum für Gesundheit und Umwelt (GmbH), Neuherberg, Germany; 4 Department of Neurosurgery, Klinikum rechts der Isar, Technische Universität München (TUM), Munich, Germany; 5 Department of Neuropathology, Klinikum rechts der Isar, Technische Universität München (TUM), Munich, Germany; 6 Department of Neurology, Klinikum rechts der Isar, Technische Universität München (TUM), Munich, Germany; 7 Department Department of Neuroradiology, Klinikum rechts der Isar, Technische Universität München (TUM), Munich, Germany; 8 Institute for Innovative Radiotherapy (iRT), Department of Radiation Sciences (DRS), Helmholtz Zentrum München, Neuherberg, Germany

## Abstract

**Background:**

Glioblastoma is usually diagnosed around the age of 60–70 years. Patients older than 65 years are frequently described as “elderly”. Several trials with monotherapy have established treatment regimens that offer therapies with reduced side effects but reduced efficacy. We analysed the outcome of elderly glioblastoma patients treated at our facility.

**Methods:**

We performed a retrospective analysis of 62 consecutive patients older than 65 years treated for a primary glioblastoma at our facility from 2009 to 2015.

**Results:**

Median age was 69.6 years (range 65.1–85.6 years); median OS of the entire cohort was 10.9 months. ECOG, MGMT and extent of resection but not age and the time from surgery to radiotherapy were associated with longer survival. Patients treated with adjuvant chemotherapy had a significantly longer survival (20.5 vs. 7.8 months). Furthermore, salvage therapies were associated with significant improved survival when compared to Best Supportive Care (22.3 vs. 8.8 months).

**Conclusion:**

Also elderly patients are likely to benefit from an aggressive treatment after primary diagnosis of glioblastoma.

## Introduction

With a median age of 64 years, glioblastoma (GBM) patients are in nearly half of cases at an age, that frequently defines them as “aged” or “elderly” [1, 2]. Generally, the present standard of care for GBM patients includes involved field radiotherapy (RT) as well as concomitant as well as 6 cycles of adjuvant chemotherapy with temozolomide (TMZ) and goes back to the study from Stupp et al. in 2005 [3]. The patient cohort, however, was limited to an age of equal or less than 70 years and a post-hoc analysis of this cohort found a negative correlation between the patient’s age and the benefit from a combined regimen [3, 4]. An anticipated increased likelihood of adverse events of TMZ in elderly patients might be one explanation for this finding [5]. Notably, several mono-institutional reports as well as data-base-studies have demonstrated that elderly GBM patients treated with standard-RT plus TMZ do have a longer survival compared to patients treated with alternative or reduced regimens, especially after extensive resection of the tumor [6–8].

As the positive effect of RT in elderly patients is not a matter of debate anymore [9], several alternative dosing-regimens have been tested in prospective trials. Exemplary, Roa and colleagues demonstrated non-inferiority of a 3 week regimen compared to the 6 week standard regimen of radiotherapy [10]. Recently, Roa and colleagues demonstrated, that also an even more hypofractionated one-week-regimen is equal-effective in elderly and frail patients compared to the formerly described mildly hypofractionated 3 week course [11]. Of note, neither of these two regimens were tested together with concomitant chemotherapy.

TMZ, on the other hand, was tested to be non-inferior to RT in elderly patients with a methylated MGMT-promotor in the NOA-08-Trial: The efficacy of RT did not depend on the MGMT-status of the treated high grade gliomas. Also this trial did not test radiochemotherapy in elderly patients [2]. This gap recently was closed by prospective data from an international phase III trial. By comparing hypofractionated RT with concomitant TMZ followed by up to 6 adjuvant cycles of TMZ, the authors demonstrated a significant advantage accompanied by a tolerable toxicity profile also for elderly patients treated with the combined regimen, independently from age and using a very inclusive paradigm [12].

While the current evidence strongly supports the role of loco-regional treatments in elderly patients, too, population based studies demonstrate a positive correlation between age and the treatment by best supportive care only, hinting to a possible under-treatment of elderly patients [7]. Hence, there must be a difference between aged frail, almost palliative patients and extremely fit and active elderly, which arguments against age only as a decision making tool.

In the present article, we report on our experience in treating elderly patients with RT and RChT and confirm that a combined modality treatment with radiochemotherapy with TMZ results in a longer survival, independently from the age but dependent from the performance status of the patients.

## Methods

### Patients

Patients with primary GBM, aged 65 years or older, starting their first course of RT between 01/2009 and 12/2015, were extracted from the prospective patient’s registry of the local department for radiation oncology at the Technical University of Munich (TUM), Germany. For all patients, treatment decisions were consented within an interdisciplinary tumor board specialised for neuro-oncologic tumors. The median age of the 62 patients was 69.6 years (range 65.1-85.6 years). Since molecular marker evaluation became a standard for all patients only recently, this information was not available for all patients: IDH mutation status was available in 32 cases (51.6%) and was negative for all of these patients. MGMT methylation was tested in 37 cases (59.6%) with 15 cases of methylated MGMT promotor (40.1%) (Table 1).

**Table 1:** Patient characteristics

Characteristics		RT (*n =* 27)	RChT (*n =* 35)
Age – years			
Median	69.6	70.4	69.3
Range	65.1–85.6	65.8–85.6	65.1–78.8
Sex – no. (%)			
Male	32 (51.6)	9 (33.3)	23 (65.7)
Female	30 (48.4)	18 (66.6)	12 (34.3)
ECOG-Score – no. (%)			
0	9	0	9 (25.7)
1	28	8 (29.6)	20 (57.1)
2	18	13 (48.1)	5 (14.3)
3	6	6 (22.2)	0
Missing	1	0	1 (2.9)
Extent of surgery – no. (%)			
Biopsy only	7 (11.3)	5 (18.5)	2 (5.7)
Subtotal resection	34 (54.8)	17 (68.0)	17 (48.6)
Gross total resection	21 (33.9)	5 (18.5)	16 (45.7)
MGMT-promotor			
Methylated – no.	15	4	11
Non-methylated – no.	22	10	12
Missing – no.	25	13	12
Time from Surgery to RT (d)			
Median	28.5	29	28
Range	12–61	12–58	14–61
RT regimen (total/ single; Gy)			
42/3	12	12	0
40.05/2.67	8	8	0
60/2 or 59.4/1.8	34	3	31
other	8	4	4
Salvage Treatment – no.	23	3	20
Radiotherapy	14	0	14
Chemotherapy	15	1	14
Surgery	14	2	12

All patients were diagnosed with operation and histological examination. In 7 cases (11.3%) patients received biopsy only; subtotal resection was performed in 34 cases (54.8%) and gross total resection could be achieved in 21 cases (33.9%). Resection status as well as evidence for postoperative ischemia was evaluated by a post-operative MRI within 48 hours after surgery. The median performance status at the onset of RT was ECOG 1 (0–3). RT was administered using 3D-conformal or intensity modulated RT in all cases and was planned with post-operative MRI, a planning MRI one week before RT and contrast enhanced planning CT with a slice thickness of 2–3 mm. The clinical target volume (CTV) consisted of the sum of the resection cavity as well as all contrast enhancing areas plus a 2 cm margin and Fluid attenuated inversion recovery (FLAIR)- or T2-hyperintense areas, plus a 1 cm margin. A margin of 5 mm was added for the Planning Target Volume (PTV). Single doses ranged from 1.8 to 3.0 Gy, total doses from 40.05 to 60.0 Gy, mean 52Gy. All patients received 5 fractions per week.

If chemotherapy was administered, patients received 75 mg temozolomide (TMZ) / m^2^ daily during radiotherapy. Adjuvant treatment was started 4 weeks after the end of RT and consisted of 150 to 200 mg/ m^2^/d of TMZ in 5 of 28 days. 6 cycles of chemotherapy were planned.

All patients were included into a strict follow-up regimen, with a first clinical visit and a first imaging study 4 weeks after RT. Clinical follow up as well as MRI-studies were repeated every 3 months. The median follow up at our institution was 6.0 months (range 0–41 months).

### Imaging

We retrospectively reviewed all imaging data and reports from our patients for the extent of the resection, the evidence of post-operative ischemia, defined by hyperintense area in diffusion weighted images (DWI, b1000) with hypointensities within spatially matched apparent diffusion coefficient (ADC) maps, and for the pattern of recurrence. We defined gross total resection (GTR) as resection of at least 99% of the contrast enhancing tumor. Subtotal resection was defined as evidence of contrast enhancing tumor after resection while resection of less than 20% of the tumor mass were defined as biopsy. Progression was defined according to the RANO-HGG criteria [13].

### Statistics

Analysis was done by SPSS v. 18. Overall as well as progression free survival were analysed with the Kaplan-Meier-method. Differences between the survival of two groups were analysed using the log-rank test (univariate statistics). Univariate cox regression analysis was used to compare categorical variables. Overall survival was defined as time from surgery to death. Progression free survival was defined as time from the start of radiotherapy to the evidence of progression according to the RANO-HGG-criteria or to death. If patients were alive at the time of our analysis (01.12.2016), survival times were censored to the date of the last follow up visit.

## Results

### Survival and pattern of recurrence

**Fig. 1:**
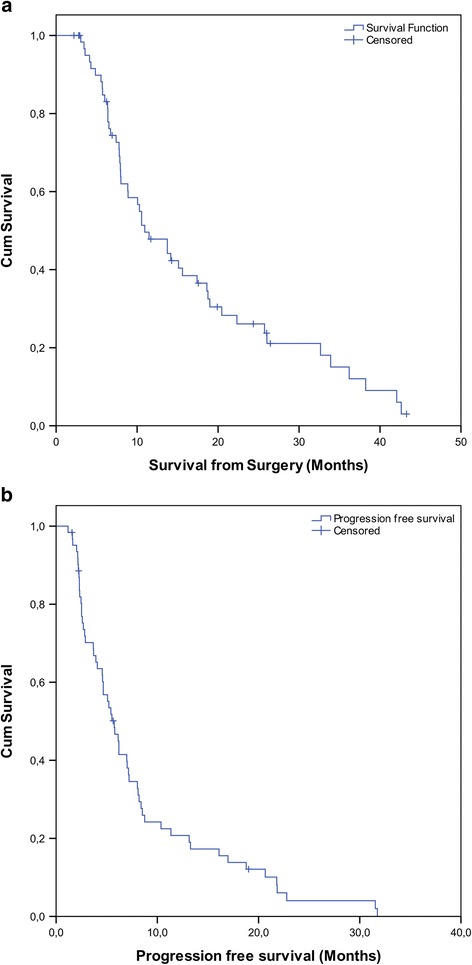
Kaplan Meier estimates of **a** overall survival and **b** progression free survival

The median overall survival of our cohort was 10.9 months (range 3.0 to 43.3; Fig. 1a). The median progression free survival was 5.7 months (range 1.2-31.7; Fig. 1b). Local recurrence occurred in 25 cases, local and distant in 10 cases and distant recurrence occurred in 4 cases. Clinical progression occurred in 7 cases, in two of these cases an MRI could not describe a specific focus. For the 5 patients remaining, MRI was not performed as the patients were not deemed to be able to undergo a salvage treatment. 12 patients died without clinical or imaging evidence for progression. At the time of our analysis, 12 patients were still alive, 1 of these patients without evidence for progression after the initial treatment, one further patient was loss of follow up.

### Treatment

Concomitant radiochemotherapy (RChT) was given to 35 patients. The median age of patients receiving a combined treatment was 69.3 years (65.1-78.8). A better ECOG was significantly associated with the initiation of adjuvant chemotherapy (*p <*0.001) as well as with the decision for a combined modality treatment (*p <*0.001). Patients receiving combined treatment generally were in a good performance status (median ECOG of 1; 0–2). All these patients received a standard fractionation regimen with single doses from 1.8 to 2.0 Gy up to a median dose of 60.0 Gy. MGMT promotor methylation was examined in 23 cases and was positive in 11 of these cases (47.8%). 1 to 9 cycles of adjuvant chemotherapy with temozolomide were given to the majority of these patients (26 of 35 patients; median No. of cycles 6) and were tolerated well. In 3 cases decision against adjuvant treatment was due to a poor performance status. Also in 3 cases, early progression occurred after RChT; one patient underwent salvage treatment, two patients were included into a best supportive care program. Two patients with severe infection during RChT decided against further chemotherapy. One patient underwent revision surgery for symptomatic radionecrosis, therefore adjuvant chemotherapy started with a delay of 3 months and the patient was not included into the adjuvant ChT group.

Twenty-seven patients were treated with RT only. The median age of this cohort was 70.4 years (65.8–85.6). The performance status was worse in this cohort with a median ECOG of 2 (1–3) and less patients received a gross total resection. Patients with mono-RT were more likely to be treated with a hypofractionated schedule with either 2.67 or 3.0 single dose up to a dose of 40.05 or 42 Gy. 2 patients received an adjuvant TMZ based chemotherapy (one patient decided against concomitant RChT, the other patient received hypofractionated treatment with 3 Gy single dose and actively decided to receive further adjuvant treatment). MGMT promotor methylation status was available for 14 patients with positive result in 4 of these cases (28.5%). For the two patients which underwent adjuvant ChT, no MGMT methylation status was available.

Twenty-three patients received some kind of salvage treatment. In more detail, 14 patients underwent re-irradiation, 15 patients received ChT for recurrent disease and 14 patients underwent surgery for recurrent disease. Patients treated for their recurrent disease had a significantly longer survival compared to patients who underwent best supportive care (BSC) at progression of their disease (mOS 8.8 vs. 22.3 months, *p <* 0.001).

### Predictors

**Fig. 2:**
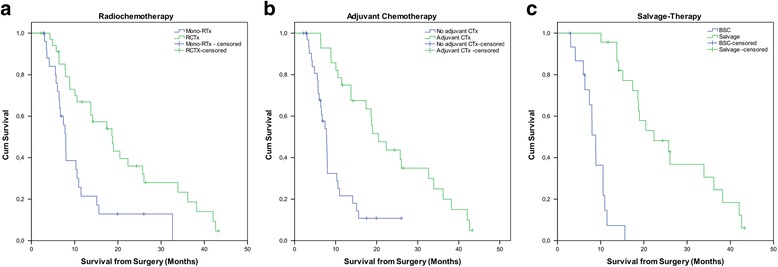
Survival stratified for Radiochemotherapy (**a**), adjuvant Chemotherapy (**b**), and e treatment (39 patients with imaging-defined recurrent GBM) (**c**)

**Fig. 3:**
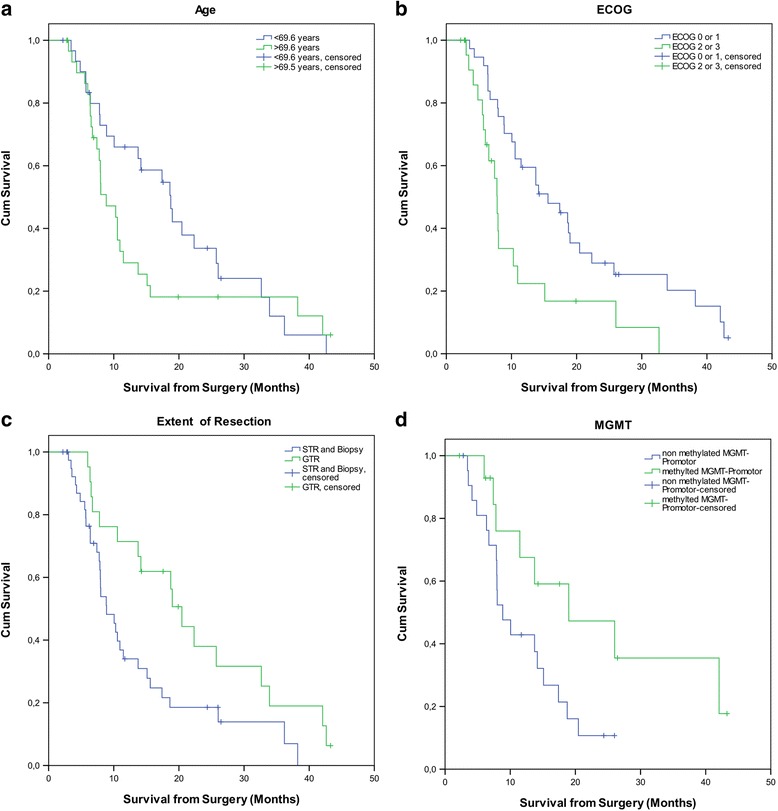
Overall survival of elderly patients stratified by Age (**a**), ECOG (**b**), Extent of Resection (**c**) and MGMT (**d**)

Treatment with chemotherapy as part of the initial treatment was the most powerful discriminator for a longer survival, with a median OS of 20.5 vs. 7.8 months (patients with vs. without adjuvant CHT; *p <* 0.001) and 18.7 vs. 7.9 months (patients with RCHT vs. patients with mono-RT; *p =* 0.002) (Fig. 2). Furthermore, ECOG (*p =* 0.008), MGMT (*p =* 0.03) and the extent of resection (*p =* 0.014) were significant predictors for a longer OS. Younger age (median 69.6 years, *p =* 0.216) and a shorter interval between surgery and the onset of RT (median 28 days, *p =* 0.82) were not associated with longer survival (Fig. 3). Postoperative ischemia was not significantly (*p =* 0.052) influencing overall survival, however, this might be a matter of numbers. All results from Cox regression analysis are summarized in Table 2.

**Table 2:** Cox regression analysis

Cox regression	HR	95% CI	*p*
Age (older vs. younger than 69.6 years)	1.44	0.81–2.54	0.216
ECOG ( 2–3 vs. 0–1)	2.35	1.28–4.34	0.008
MGMT (negative vs. positive)^a^	2.63	1.04–6.66	0.03
Extent of resection ( GTR vs. STR & Biopsy )	0.47	0.25–0.876	0.014
Ischemia (ischemia vs. no ischemia)^b^	1.94	0.98–3.82	0.059
Time from surgery to RTx (earlier vs. later than 28 days)	1.07	0.61–1.89	0.816
Adjuvant CHT vs. No Adjuvant CHT	0.24	0.13–0.47	<0.001
RCHT vs. RT	0.38	0.21–0.69	0.002
Treatment for recurrent disease (Treatment vs. BSC)^c^	0.06	0.02–0.17	<0.001

^a^only patients with measured MGMT methylation; ^b^only patients with postoperative imaging including DWI b1000 and ADC; ^c^only patients with recurrence diagnosed by imaging

**Fig. 4:**
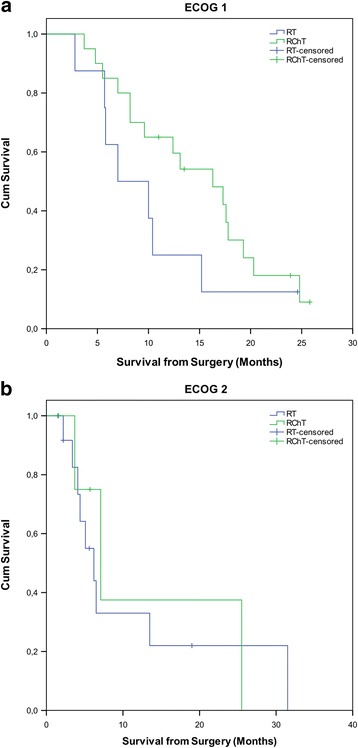
Kaplan Meier estimares for patients undergoing RT and RChT. In patients with ECOG 1 (**a**), median OS 7 vs. 16.3 months. In patients with ECOG 2 (**b**), median OS was 6.2 vs. 7.2

Importantly, age was not significantly related to a worse ECOG (*p =* 0.11; Chi-square test), but it was related to the initiation of radiochemotherapy (*p =* 0.001; Chi-square test) and the prescription of an adjuvant chemotherapy (*p =* 0.005; Chi-square test). Furthermore, we asked whether the subgroup patients with an ECOG of 1 or 2 would benefit from RChT or not. The median OS of ECOG 1 patients was 7 and 16.3 months with RT and RChT, respectively (Fig. 4a). The difference was not significant (*p =* 0.174), however, this is most likely due to the low patient number of this subgroup. In comparison to this, the median OS of patients with an ECOG of 2 was 6.2 and 7.2 with RT and RChT (Fig. 4b). Also this difference was not significant (*p =* 0.774).

## Discussion

GBM almost always leads to the loss of independence by increasingly developing disabilities throughout the course of disease; the loss of independence is mostly due to progression of the disease [14]. RT, chemotherapy as well as combined modality treatments have shown to prolong the progression free survival and to increase the OS, too [2, 15, 16]. Based on reports about increased toxicities of either of these treatments in elderly patients, mono-therapeutic regimens have emerged [2, 10, 11]. All of these aim on minimizing the burden by the treatment, and, all of them have demonstrated mOS between 6.4 and 9.6 months in elderly people [2, 10]. Inclusion criteria of these trials were mostly based on age (65 years or older) and a modest to good performance status (Karnofsky Performance Score (KPS) of at least 60% or ECOG of 2 or less).

The median age of the NOA-08 trial, a trial comparing chemotherapy to RT in elderly patients, was 71 vs. 72 years, the median OS was 8.6 for TMZ and 9.4 months for RT, *p =* 0.033. The trial also analysed the impact of MGMT promotor methylation and described the predictive value for MGMT for the efficacy of TMZ. Similar results were reported from the Nordic Trial, which furthermore reported a small but significant positive effect of either TMZ or hypofractionated RT to a standard fractionated RT [17]. The trial included patients with an age of 60 years or older, the difference described above was more pronounced within the group of patients older than 70 years. Rao et al. randomized patients with a minimum age of 60 years and a mean age of 72.4 and 71 years to receive either a 6 week normofractionated or a 3 week hypofractionated regimen. Both groups had a median KPS of 70%. The trial was closed earlier due to high similarity between the two arms. The trial demonstrated equal efficacy of both dosing schemes, with a median OS of 5.1 and 5.6 months. Notably, Gross Total Resection (GTR) was achieved in only 4.2 and 14.6% of the cases, almost 40% received biopsy only [10]. A mono-institutional report from Ontario, Canada, reported about hypofractionated RT with and without concurrent TMZ. GTR was achieved in one third of the patients. The median survival was superior for the mono-RT group (6.9 vs. 9.3 months), yet the difference was not significant. Similar to our results, also elderly patients had a significant benefit from salvage therapies (5.7 vs. 13.3 months) [18]. Already in 2008, Combs et al. demonstrated the efficacy and safety of RChT for patients older than 65 years. This cohort had a median survival of 11 months, a subgroup with gross total resection had a median survival of 18 months [8]. Only recently, results from the randomized phase III EORTC 26062–22061 / TROG 08.02-Trial were presented by Perry and colleagues. 562 patients were randomized between a hypofractionated RT up to 40.05 Gy alone or in combination with concurrent TMZ. In opposite to the retrospective data from Cao et al., this trial showed a significant advantage for the combined modality treatment (7.6 vs 9.3 months). Importantly, also patients with non-methylated MGMT-promotor benefited from concurrent radiochemotherapy [12]. Unfortunately, the article did not report whether also patients with ECOG 2 did profit from the combined modality treatment. In our cohort, albeit not significant, patients with an ECOG 1 did profit more than patients with ECOG 2. In our view, the radiotherapy treatment regimen could be an important reason for this difference. Hypofractionated radiotherapy was shown to be equally effective in elderly and frail patients not eligible to standard fractionated treatment [10, 11, 17], but concerns about the long term safety have been raised [19]. On the other hand, nether safety nor efficiency of hypofractionated and standard-fractionated RChT have been compared directly. Taken that in hypofractionated treatments the concomitant chemotherapy time it cut in half, medically fit patients (ECOG 0 and 1) treated according to the Perry-Study can be considered to be under risk of being undertreated. A standard fractionated RChT therefore should be deemed to be the standard of care for elderly patients with a good performance status. This concepts has been published previously, stressing the necessity of proactive treatment in medically fit patients above age 65 years [8].

We also analysed the survival of patients after diagnosis for objective progressive disease. Patients which were treated for recurrent GBM, either by surgery, re-irradiation or chemotherapy, did have a longer survival compared to patients receiving best supportive care (BSC) in this situation. Prospective trials comparing BSC and re-irradiation or any other salvage-strategy in elderly patients are scarce; only one article analysed this topic and concluded, that salvage treatment for recurrent GBM could be beneficial [20]. This comparison as well as our analysis are influenced by a selection bias, as patients with a better performance score are more likely to undergo a salvage treatment than patients with a poor OS. Concerning re-irradiation, an interval of less than 6 months between the first course of radiotherapy and progressive disease is usually believed as to short to undergo re-irradiation. As RChT can result in progression free survival of more than 6 months in elderly patients, especially when MGMT promotor hypermethylation is present [12], re-irradiation could become more frequent. Further studies, especially on the safety and efficacy of re-irradiation, are therefore highly recommended.

Our data underline the role of an intensive early treatment as well as of salvage treatments for older patients. Especially patients treated with standard-fractionated RChT as well as adjuvant chemotherapy had a median OS that was similar to younger patients. This is in-line with the conclusion from a SEER-based analysis from 2015 [7]. Reasons for this might be the good physical status of patients within this cohort as well as the high amount of patients with methylated MGMT promotors. As the performance score but not age significantly related to the treatment decision and to the outcome, monotherapy should only be considered for patients older than >70 years and presenting with a lower performance score. This algorithm is also in line with the resent guidelines for the treatment of GBM from the American Society of Clinical Oncology (ASCO) [21]. Notably, when reviewing earlier studies or studies from other geographic areas, the huge geographic differences of life expectancies have to be taken into account. Exemplarily, the life expectancy within the western world approximately increases every 20 years by 5 years, currently reaching an average life expectancy of 80 years, compared to an average life expectancy of 66 years in India.

## Conclusion

Combination of RT and chemotherapy in elderly patients, independently of fractionation, has a good efficacy also in elderly patients and should be considered even in higher age but with taking the performance status into account. Therefore, treatment decision should be made based not only on age in order to prevent undertreatment in elderly patients.

## Abbreviations

ADC: Apparent diffusion coefficient
BSC: Best supportive care
ChT: Chemotherapy
CTV: Clinical target volume
DWI: Diffusion weighted images
ECOG: Eastern cooperative oncology group
FLAIR: Fluid attenuated inversion recovery
GBM: Glioblastoma
GTR: Gross total resection
GTV: Gross tumor volume
IDH: Isocitrate dehydrogenase
MGMT: O6-methylguanine-DNA-methyltransferase
mOS: Median overall survival
OS: Overall survival
PFS: Progression free survival
PTV: Planning target volume
RChT: Radiochemotherapy
RT: Radiotherapy
TMZ: Temozolomide
